# Current Perspectives on Role of MSC in Renal Pathophysiology

**DOI:** 10.3389/fphys.2018.01323

**Published:** 2018-09-20

**Authors:** Min Fan, Jing Zhang, Hong Xin, Xiaozhou He, Xuemei Zhang

**Affiliations:** ^1^The Third Affiliated Hospital of Soochow University, Changzhou, China; ^2^Department of Pharmacology, School of Pharmacy, Fudan University, Shanghai, China

**Keywords:** mesenchymal stem cell, renal fibrosis, regenerative therapy, homing, autocrine, paracrine, pro-fibrotic

## Abstract

In the course of the development and worsening of kidney disease, the treatments available are expensive and may cause adverse effects such as immune rejection, inadequate renal resources, or post-operative complications. Therefore, there is an urgent to develop more effective treatments. The advent of mesenchymal stem cells (MSCs) represents a new direction in this context. The current use of MSCs for the treatment of kidney disease has mostly involved experimental studies on animals and only a few clinical trials have been conducted. This review focused on the mechanisms of MSC involvement from different sources in the improvement of renal pathophysiology in recent years. These mechanisms include homing to damaged kidney tissue, and differentiating into or fusing with the innate cells of the kidney. The paracrine or endocrine action through secreting protective cytokines and/or releasing microvesicle from MSCs also plays a critical role in amelioration of kidney disease. With modern engineering technology like microRNA delivery and a combinational therapy approach such as reduction of renal fibrosis in obstructive nephropathy with MSCs and serelaxin, MSC may make great contribution to the improvement of renal pathophysiology. However, the therapeutic effects of MSC are still controversial and several problems remain unsolved. While it is too early to state that MSCs are useful for the treatment of renal diseases in clinic, it is thought that solutions to the existing problems will enable effective modulation of the biological characteristics of MSCs, thereby providing new and effective approaches for the treatment of renal diseases.

## Introduction

Mesenchymal stem cells (MSCs) are a type of pluripotent stem cells of the mesodermal origin that play a very important role among different stem cells. Because of their strong proliferative capacity and the potential of multi-lineage differentiation, MSCs can easily differentiate into osteoblasts, cartilage, or adipocytes under suitable induction conditions *in vivo* or *in vitro*. They also have the capacity to differentiate into cardiac, liver, endothelial, hematopoietic, nerve, islet, and several other cell types and retain their differentiation potential even after continuous sub-culturing and cryopreservation (Camassola et al., 2012) thereby providing a new source for the clinical treatment of various diseases. In addition, because of the wide range of sources for their isolation; ease of isolation, culture, expansion, and purification; low immunogenicity, desirable re-implantation characteristics (Semedo et al., 2009), and limited ethical issues, MSCs have great potential applications in tissue engineering, gene therapy, and immunotherapy.

The prevalence of kidney disease is estimated to be 8–16% worldwide. With an aging population, and rising levels of hypertension, diabetes and obesity, renal diseases pose an increasing burden on public healthcare. Two million people worldwide are currently on renal replacement therapy (RRT), dialysis or have a renal transplant. However, a greater number of patients died due to the inadequate availability of therapies and skewed treatment toward affluent countries with access to healthcare. It is therefore imperative that we develop new strategies to identify those at high risk of progressive kidney disease and to discover new therapies to slow the rate of disease progression in these individuals.

**FIGURE 1:**
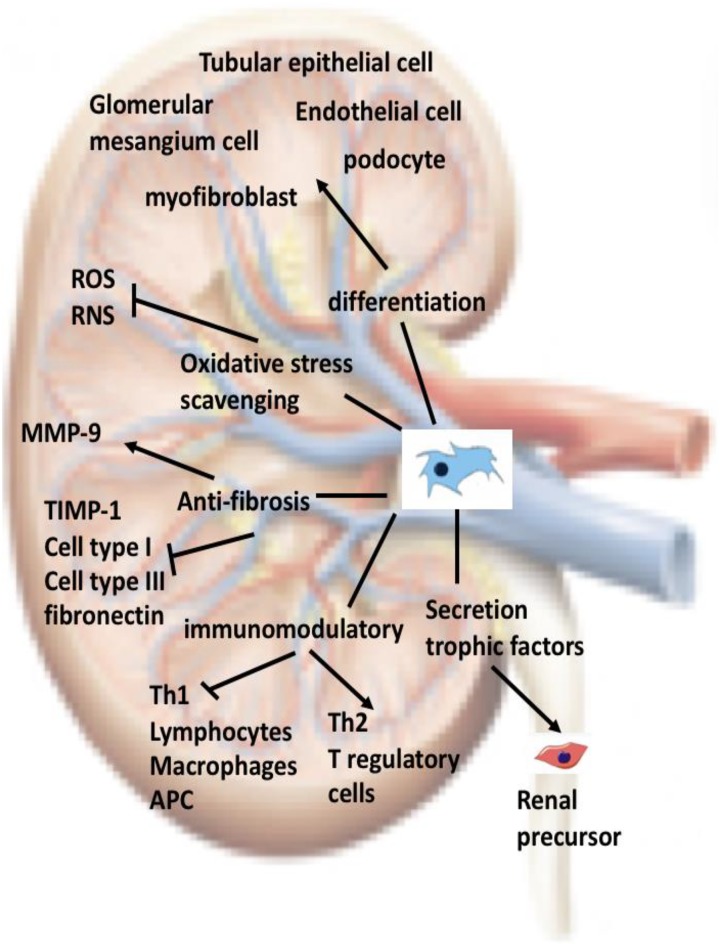
Current strategies for the treatment of kidney diseases with MSC: Once administered into an individual, MSCs will circulate into the bloodstream. In a damaged kidney, MSCs will cross the endothelium, reside in the parenchyma, and then migrate to the injured areas. MSCs contribute to renal tissue regeneration through at least one of the mechanisms depicted in the figure. ROS, reactive oxygen species; RNS, reactive nitrogen species; APC, antigen presenting cell.

Although several clinical trials using human autologous or allogeneic stem cells have been conducted for the treatment of human diseases, the current use of MSCs for the treatment of renal disease has mostly involved experimental studies on animals and only a few clinical trials have been conducted. It is still too early to state that MSCs are commonly used in the treatment of kidney disease. Several problems remain unsolved, involving (1) the identification of specific tissues, organs, or individual-derived MSCs that are most suitable for the treatment of kidney disease; (2) the number of MSC transplantations and of MSCs to be transplanted to achieve sufficient inhibition or RF reversal; (3) the stage of kidney disease progression at which MSCs can be used; (4) addressing different causes of disease with different treatment strategies, including gene therapy using MSCs as carriers; and (5) long-term effects and safety of MSCs after transplantation. However, current strategies for the treatment of kidney diseases with MSC have made contributions to modern medicine (**Figure 1**).

## Sources of MSCs

### Bone Marrow

Mesenchymal stem cells of different origins and stored at different sites, such as the bone marrow and circulatory system, can be released from storage to participate in kidney injury repair under certain conditions (Duffield and Bonventre, 2005).

### Pericytes

Based on the current understanding, the renal papilla (Oliver et al., 2004) or the renal Bowman’s capsule (Sagrinati et al., 2006) is considered a stem cell “niche” in mature kidneys (Chen et al., 2008). Peritoneal cells have been relatively less understood for several years, but they have recently been well studied as a relevant cell population in renal biology and pathophysiology. Pericytes are stromal cells that support the vasculature, and MSCs are a type of pericytes. In the kidney, pericytes have been reported to play a key role in the regulation of angiogenesis, in the renal medulla, and in cortical blood flow, in addition to serving as interstitial myofibroblasts (MFBs) in RF. They interact with each other via different signaling pathways, and their activation and detachment from the capillaries after acute or chronic kidney injury may be the key to driving CKD progression. In contrast, pericytes may also act as a local population of stem cells during kidney homeostasis, replenishing the differentiated interstitial and vascular cells lost during aging (Kramann and Humphreys, 2014).

### Allogeneic

Exogenous MSCs are obtained through vascular or local injection (Peired et al., 2016). Allogeneic stem cell transplantation involves transferring stem cells from a healthy person (donor) to a recipient following high-intensity chemotherapy or radiation. Allogeneic stem cell transplantation is used to treat patients with a high risk of relapse who do not completely respond to treatment or patients who relapse after a prior successful treatment. Allogeneic stem cell transplantation is considered a high-risk procedure. The high-conditioning regimens severely or completely impair the recipient’s ability to produce stem cells, and they will likely experience side effects on the days of high-dose conditioning radiation or chemotherapy treatment.

## Mechanisms of MSC Involvement in the Improvement of Renal Pathophysiology

### The First Functional Mechanism of MSCs Involves Homing to Damaged Kidney Tissue and Differentiating Into or Fusing With the Innate Cells of the Kidney

#### Differentiation Into the Innate Cells of the Kidney

Ito et al. (2001) introduced green fluorescent protein (GFP)-labeled mouse BMSCs into wild-type mice with anti-Thy1 glomerulonephritis via intravenous injection and found that 7–8% of the mesangial cells in reconstructed globules were GFP-positive, CD45-negative, and Thy1-positive, thereby confirming that MSCs can differentiate into mesangial cells *in vivo*. Zou et al. (2016) transplanted male rat BMSCs into CAAN female rats; 4 weeks later, double-positive cells were found in their kidney tissue using Y chromosome fluorescence *in situ* hybridization and immunofluorescence staining with EC-marked antigen CD34, indicating that MSCs can differentiate into EC *in vivo*. Further, Yokoo et al. (2005) injected human BMSCs into pre-implantation mouse embryos. After 48 days of embryo culture, embryos and kidney were isolated and their cells cultured for 6 days. The results showed that not only could human BMSCs differentiate into various kidney-specific cells, including podocytes, RTECs, and stromal cells, but also they could form complete nephrons. The most recent finding by Wang et al. (2016) shows that the use of potentially breakthrough miRNA therapy has been hampered by a lack of an understanding of the targeting of miRNAs to damaged kidneys. Using bioluminescence imaging of UUO mice, they found that MSCs overexpressing miRNA-let7c (miR-let7c-MSCs) selectively home the injured kidney compared with non-targeted control (NTC)-MSCs.

From this body of research, we know that following kidney injury due to inflammation, ischemia, or hypoxia, autologous or exogenous MSCs are prone to preferentially cross the vascular endothelium, return to the damaged kidney tissue, and differentiate into kidney cells under the influence of various factors.

#### Fusion With the Innate Cells of the Kidney

Cell fusion refers to a phenomenon that naturally occurs during tissue growth and regeneration in which two or more cells of the same or different types combine into one and show mixed genotypes and phenotypes. MSCs can interact with cardiomyocytes, hepatocytes, and Purkinje cells; therefore, it has been speculated that MSCs can fuse with the innate cells of the kidney, although this remains a controversial issue. Held et al. (2006) have found that nearly 50% of proximal tubule epithelial cells could be replaced by bone marrow-derived cells following the transplantation of bone marrow from wild-type normal female mice into fumarylacetoacetate hydrolase-deficient transgenic male mice with severe tubular necrosis. Most of the repair is the result of the fusion of bone marrow-derived and tubular cells, rather than direct trans-differentiation, even when the fusion involves hematopoietic-derived myelomonocytic cells instead of MSCs. Another strong evidence is a study by Ozbek et al. (2015) on the protective effect of the vascular endothelial growth factor (VEGF) and MSC transplantation on renal injury induced by UUO. Stem cells transplanted into the kidneys prevented RF while preventing UUO from causing epithelial–mesenchymal transitions (EMTs).

Several studies have demonstrated that MSCs can fuse autonomously with existing host cells, which may explain the ability of bone marrow-derived stem cells to generate cells with non-hematopoietic lineages. Bone marrow-derived MSCs can fuse with hepatocytes, cardiomyocytes, and Purkinje cells, resulting in heterozygous genotypes and phenotypes. Various different non-hematopoietic and mesodermal cell types may originate from the fusion of stem cells with cells of various lineages. In the case of chronic renal injury and continuous gene selection, bone marrow-derived stem cells or MSCs can be induced to fuse with renal tubular cells to restore renal function.

### The Second Functional Mechanism of MSCs Involves the Promotion of the Repair of Damaged Kidneys Through Paracrine or Endocrine Action

This comprises two types of processes as follows:

#### Secretion of Protective Factors to Promote Endogenous Repair

Mesenchymal stem cells can exert a reparative effect on kidney injury through a series of protective factors secreted by endocrine/paracrine mechanisms, including anti-inflammatory factors, insulin-like growth factor-1 (IGF-1), VEGF, hepatocyte growth factor, soluble factors, growth factors, and chemokines (Nigam and Lieberthal, 2000).

Togel et al. (2005) transplanted fluorescent protein-labeled MSCs through the left carotid artery into a rat model immediately or 24 h after ischemia–reperfusion injury and observed a significant improvement in the renal function, an acceleration of cell proliferation, and a slowing of apoptosis after transplantation. After 24 h, the expression levels of TNF-β, IL-1B, and INF-1 were significantly decreased, while those of the anti-inflammatory cytokines TGF-β, IL-1β, IL-10, and Bcl-2 increased. In the early stage, MSCs could be detected in the glomerular and renal microvasculature. However, no transplanted MSCs differentiated into RTECs within 3 days after transplantation. Therefore, the repair effect of MSCs on the kidney is not directly induced by target cells but via a complex paracrine mechanism to reduce the inflammatory reactions in the injured kidneys; this mechanism plays an important role, especially during the later period of repair.

In another study, Imberti et al. (2007) co-cultured MSCs with proximal tubular epithelial cells (PTECs) impaired by CSP *in vitro* and found that the MSCs secreted large amounts of IGF-1 to promote renal proliferation. However, when co-cultured with IGF-1-depleted MSCs, the CSP-impaired PTECs showed decreased proliferation and increased apoptosis. Further, when mice subjected to CSP-induced acute kidney injury were transplanted with IGF-1-depleted MSCs via tail vein, renal protection and tubular structure was found to be limited, suggesting that MSCs promote the repair of tubular cells via the secretion of IGF-1. Togel et al. (2009) transplanted VEGF gene-deleted MSCs via the left internal carotid artery in rats and found that renal protection significantly decreased, suggesting that VEGF is the most important renal protection factor secreted by MSCs; VEGF stimulates hyperplasia in tubular peripheral capillaries and protects the renal epithelial cells from injury, thereby protecting glomerular function and promoting tubular regeneration.

Mesenchymal stem cells have shown anti-inflammatory and anti-fibrotic effects in rodent CKD models, and relevant animal experiments have now been extended to other mammals. Quimby et al. (2016) used chronic tubulointerstitial nephritis-induced cats as a characterized model for further evaluation of the effectiveness of allogenic MSCs in the treatment of mammalian kidney disease.

At present, many scholars believe that MSCs exert their therapeutic effects through the paracrine effects of multiple cytokines and microvesicles (MVS), and that this mechanism is more important than that of MSCs directly transforming into injured renal innate cells. The paracrine effects of injected MSCs may include activation of endogenous renal cells, promotion of angiogenesis, inhibition of oxidative stress, reduced apoptosis, inflammation, and RF.

#### MV-Mediated Genetic Information Transfer

Microvesicles, also known as micro-particles, are multi-functional vectors comprising membrane-wrapped vesicles with diameters ranging from approximately 0.05–1 μm that are released from cell membranes during outward budding and cell fission (Cocucci et al., 2009) and contain multiple components, including cell membrane receptors, adhesion molecules, signaling proteins, and functional genetic information due to mRNAs and miRNAs (Muralidharan-Chari et al., 2010). Unlike traditional MSCs that secrete soluble factors, growth factors, and chemokines through endocrine or paracrine secretion mechanisms, MVs released by MSCs exist in their conditioned medium, and substances in MVs are not only directly involved in the regulation of cellular biological activity but also function as a cellular stress signal in target cells, transmitting signals via specific receptor-ligand binding or MV-cell fusion and regulating inflammatory immune responses and repair mechanisms (Camussi et al., 2010).

Several preclinical studies have shown that the administration of exogenous MSCs prevents kidney damage and promotes kidney recovery through a complex series of mechanisms, particularly via the regulation of the immune system and release of paracrine factors and MVs (Wu et al., 2018).

Quesenberry and Aliotta (2008) found that the stem cell phenotype can undergo reversible changes during the cell cycle, and the microenvironment in which the stem cells are located plays a key role in changes in cellular phenotype and plasticity. MV-mediated intercellular genetic information transfer after the release of MVs from damaged cells regulates microenvironment changes, thereby initiating interaction between stem cells and their microenvironment and prompting stem cell recoding to attain the characteristics of damaged cells. In contrast, MVs released by stem cells are taken up by surviving cells at the injury site and then re-enter the cell cycle, causing the self-repair of cells.

Further, Wang et al. (2015) determined differences in the miRNA expression in age-related BM-MSC-MVs and the correlation between miRNA expression and the RF process. In aged rats, BM-MSC-MVs had a lesser inhibitory effect on EMT induced by TGF-β1 than did HK2 cells in young rats. In addition, downregulated miR-133b-3p and miR-294 in BM-MSC-MVs of aged rats significantly inhibited TGF-β1-mediated EMT in HK2 cells, suggesting that MSC therapy is more effective in elderly fibrotic kidneys.

Mesenchymal stem cell-derived extracellular vesicles (EVs) are considered effective mediators of renal injury repair. Angiogenesis is considered an important step in tissue regeneration. However, the proangiogenic effect of MSC-EVs on ischemia–reperfusion-induced renal injury and its underlying mechanisms remain to be determined. Zou et al. (2016) have demonstrated that human MSC-EVs can protect against ischemia–reperfusion-induced renal injury by promoting HIF-1α-independent angiogenesis while promoting VEGF and RNA delivery related to angiogenesis. Chen et al. (2017) have determined that MVs derived from human WJ-MSCs improve IR-induced RF via the Erk1/2 signaling pathway and a G2/M cell cycle block.

**FIGURE 2:**
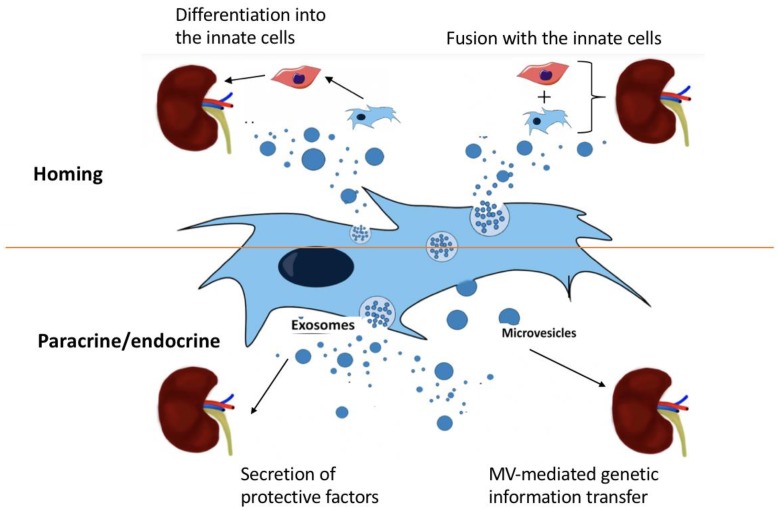
The four potential mechanisms of MSC treatment on kidney disease: (i) differentiation into innate cells, (ii) fusion with innate cells, (iii) secretion of protective factors by exosomes, and (iv) MV-mediated genetic information transfer.

Microvesicles released from the surface of activated cells are relatively larger (100 nm to 1 μm diameters) than Exo, which are smaller membrane fragments (30–90 nm diameters), that originate from the endosomal compartment after fusion of secretory granules with the plasma membrane. Systemic injection of either MVs or cells promoted a comparable regenerative program in damaged renal tissue. This study characterized the transcripts present in MVs and demonstrated the shuttling *in vitro* and *in vivo* of two mRNAs encoding proteins involved in proliferation. Furthermore, another study reported a new mechanism underlying the beneficial effect of BM-MSC derived Exo on proximal tubular cells exposed to cisplatin. The repair of cisplatin-damaged proximal tubular cells resulted from a combined trophic effect of IGF-1 released by BM-MSC and the transfer of mRNA of the corresponding IGF-1 receptor via Exo, which potentiates tubular cell sensitivity to the growth factor. The use of MVs derived from MSC as a strategy to enhance survival in Acute Renal Failure (ARF) and to protect against RF has been proposed (**Figure 2**).

### The Third Mechanism of the Use of MSCs Involves the Application of Modern Engineering Technology

MicroRNAs (miRNAs) are short single-stranded RNAs that are involved in the post-transcriptional regulation of gene expression and play a role in CKD pathogenesis. Recent evidence suggests that miRNAs reduce RF by modulating collagen and accumulating related targets; however, the development of miRNA therapy is hampered by the lack of targeted and sustainable systemic delivery of miRNAs. MSCs are present in endogenous immune-related and inflammatory sites and release growth factors to regulate the immune system, alter macrophage polarization, and protect and repair the kidneys while overcoming toxicity, mutations, and low efficiency; thus, MSCs provide an effective mechanism of miRNA delivery. Designing MSCs that express or overexpress miRNA released by exosomes can enhance their function. Clinical studies have used cancer- and CKD-associated MSCs, indicating that the combination of miRNAs and MSCs may provide a novel cell-based approach for the treatment of CKD (Yao and Ricardo, 2016).

Wang et al. (2016) showed that the potent gastric anti-secretory function of engineered MSCs enables selective transport of miR-let7c-MSCsto damaged renal cells and provides a pathway for miRNAs in MSCs to ameliorate kidney disease. Compared with control MSCs, miR-let7c-MSCs can reduce renal damage and significantly reduce the gene expressions of collagen IVα1, metalloproteinase (MMP)-9, transforming growth factor (TGF)-β1, and TGF-β type 1 receptor (TGF-βR1) in UUO kidneys in mice. *In vitro* analysis has confirmed that the transformation of miR-let7c from miR-let7c-MSCs occurs via secreted exosomal uptake, which was visualized in NRK52E cells using cyc3-labeled pre-miRNA-transfected MSCs with or without the exosomal inhibitor GW4869. The upregulated expression of fibrotic genes in NRK52E cells, induced by TGF-β1, was repressed by the addition of isolated exosomes or indirect co-culture with miR-let7c-MSCs compared with the culture with control MSCs. Further, the co-transfection of NRK52E cells with the 3′-UTR of TGF-βR1 confirmed that miR-let7c attenuates TGF-β1-driven TGF-βR1 gene expression.

### The Fourth Mechanism of the Use of MSCs Involves a Combinational Therapy Approach

In addition to the use of engineering technology to cause a relevant transformation of MSCs, various combinations of treatment have emerged as effective options. Huuskes et al. (2015) have demonstrated that combination therapy with MSCs and serelaxin effectively reduced RF in obstructive nephropathy. The antifibrotic factors combined with MSCs improved RF, including tissue repair and renal tubular epithelial damage repair, compared with either single treatment. In addition, the combination of macrophage infiltration and MFB localization also stimulated a decrease in MMP-2. Huuskes et al. (2015) have found that BMSCs combined with a low dose of FK506 can lead to improved immunosuppressive function. BMSCs may accelerate the recovery of renal glomerular filtration and renal tubular reabsorption during the early stages after renal transplantation. Due to the long-term effect of their combination with low doses of FK506, BMSCs can effectively reduce chronic degenerative changes in renal grafts and toxic effects of calcineurin inhibitors on the structure of renal grafts, thereby improving the long-term survival of renal allografts.

### Clinical Trial of MSCs for Fibrotic Kidney Disease

Mesenchymal stem cells present unique properties depending on their environment and tissue of residence. Several lines of evidence support that TGFβ and other molecules, which were previously believed to play a well-defined role in fibrosis across multiple organs, are less prominent than other tissue-specific mechanisms.

Clinical trial failures in fibrotic kidney disease have resulted in the questioning of the long-standing hypothesis that TGFβ is the central regulator of fibrosis across multiple organs and diseases. A phase 2 trial of pan-TGFβ blockade in diabetic nephropathy using a blocking antibody resulted in no impact on any disease end point, despite progressive fibrosis being strongly implicated in the disease pathogenesis (ClinicalTrials.gov NCT01113801). A phase 2 study of TGFβ blockade in focal and segmental glomerulosclerosis has not been continued (ClinicalTrials.gov NCT01665391). However, novel pathways, including the previously mentioned Hedgehog signaling pathway, are emerging as promising therapeutic avenues for the treatment of kidney fibrosis. GANT61, a compound that inhibits GLI activity in the nucleus, specifically blocks the proliferation of Gli1+ MPCs and MFBs *in vitro*. *In vivo* treatments using two separate compounds that reduce GLI protein expression—darinaparsin, an anti-neoplastic drug approved by the FDA, and GANT67—resulted in reduced collagen deposition and αSMA expression in an experimental model of kidney fibrosis (Kramann et al., 2015). Neither of the molecules inhibited tubular epithelial cell proliferation, thereby highlighting the specificity of the Hedgehog pathway to the mesenchymal compartment of the kidney.

The combination of transgenic mouse models, which are instrumental in studying the *in vivo* mechanisms underlying fibrosis and preclinical testing of antifibrotic compounds, allows the evaluation of emerging antifibrotic therapies with higher accuracy. Limitations arising from differences between mice and human biology and from the etiology of the fibrogenic process in experimental models versus that in human diseases, however, preclude direct extrapolation of experimental data and highlight the need for careful interpretation of any results obtained with those models.

Meanwhile, for the treatment of Chronic Renal Failure, there is a clinical trial to investigate the biological characteristics of adipose tissue-derived mesenchymal stem cells (AMSCs) and its treatment effects on chronic renal failure. This study aims to investigate the biological characteristics of AMSCs and its effect on oxidative stress, inflammation, and mitochondrial damage. It is intended to use blood oxygen level-dependent magnetic resonance imaging (BOLD-MRI), emission computed tomography (ECT) and enhanced magnetic resonance imaging to monitor renal oxygenation, tissue perfusion and renal function.

## Controversy Regarding the Effects of MSCs

One controversy in the last year revealed adverse therapeutic effects of MSCs on the treatment of renal diseases such as RF (El Agha et al., 2017). MSC-like cells are increasingly being considered the predominant fibrosis-associated fibroblasts in various organs. These findings are mainly based on genetic lineage tracing, which is the gold standard for studying the response of cell fate to pathological stimuli. Chronic exposure to pro-inflammatory and pro-fibrotic cytokines may cause damage to MSC-like cells by triggering epigenetic modifications and activating matrix-related genes, resulting in their involvement in MFB differentiation and fibrosis development. Unfortunately, anti-inflammatory agents do not prevent the progression of fibrosis in patients. In contrast, the targeting of MSC-like cells to organs is challenging because these cells not only exacerbate fibrosis but also contribute to tissue homeostasis by preserving stability and integrity. In addition, Ninichuk et al. (2006) has found that although weekly injection of MSCs prevents the loss of capillaries surrounding perirenal tubules but could not delay the rate of renal failure and ensure the survival of COL4A3-deficient mice.

Another controversy is what roles of autophagy induced by MSC play in kidney disease. Induction of autophagy and its contribution to fibrotic diseases has been suggested in the lung, liver, and heart. The pathological roles of autophagy in fibrosis in those organs vary greatly depending on the type of cells or tissues and pathological conditions. Evidence thus far regarding autophagy in kidney fibrosis is mainly from the studies using UUO and TGF-β models. Under these conditions, involvement of autophagy in either tubular atrophy or degradation of collagen has been suggested, which apparently contribute oppositely to the pathogenesis of renal fibrosis. Persistent activation of autophagy may contribute to tubular atrophy and thereby promote kidney fibrosis. Alternatively, autophagy can prevent fibrosis by mediating intracellular degradation of excessive collagen. Further research should focus on the regulation of autophagy in kidney injury and repair as well as the role of autophagy in renal fibrosis following AKI.

## Conclusion and Prospects

The current understanding of the biological characteristics of MSCs is fairly extensive, and the relationship between MSCs and renal diseases, including fibrosis, are becoming increasingly clearer and more systematic. Further exploration of the use of MSCs for the treatment of such conditions and the development of related technologies and mechanisms will hasten the introduction of this treatment strategy into clinical settings and its more effective application in practice. Currently, controversies surrounding the use of MSCs in this context include a possible tumor formation or malignancy, the increased probability of infection, and the duration of immunosuppression due to MSCs. Notable advantages of MSCs include their capacity to be induced into various cell types and tissues and their ability to assume the corresponding cellular morphology and phenotype and perform relevant cellular functions (Camassola et al., 2012).

Auto-MSCs are not suitable for the treatment of acute diseases because extraction is time-consuming. Due to their immune suppression properties and low immunogenicity compared with other cell types, the implantation of allogeneic MSCs (allo-MSCs) maybe more appropriate. Despite inconsistent conclusions about the therapeutic effects of allo-MSCs, they remain a promising option in immunosuppressive and tissue repair therapy.

To date, we have failed to obtain consistent results on the immunogenicity and protective effects of allo-MSCs. Thus, the following issues must be addressed in future research. First, what immune molecules and cells are involved in the potential immune response? Second, what is the dynamic fate of implanted allogeneic adult stem cells (ASCs) including elimination by recipients, being maintained in a stem cell state, or differentiating into various cell types? It would be useful to assess the *in vivo* efficiency of allo-MSCs compared with that of auto-MSCs. Third, the factors that influence therapeutic effects of MSCs and how they affect the results remain unclear. Finally, strategies to enhance the consistency and efficacy of allo-MSCs as a cell-based therapy should also be investigated in inflammatory diseases for tissue repair.

While it is too early to state that MSCs are useful for the treatment of kidney disease, it is thought that solutions to the existing problems will enable effective modulation of the biological characteristics of MSCs, thereby providing new and effective approaches for the treatment.

## Ethics Statement

The study was approved by the institutional review board (CWO) of Fudan University, China.

## Author Contributions

XH contributed to the conception of the study. MF contributed significantly to analysis and manuscript preparation. JZ wrote the manuscript. XZ helped perform the analysis with constructive discussions. HX approved the final version.

## Conflict of Interest Statement

The authors declare that the research was conducted in the absence of any commercial or financial relationships that could be construed as a potential conflict of interest.
